# The Association between the Serum C-Peptide Level and Bone Mineral Density

**DOI:** 10.1371/journal.pone.0083107

**Published:** 2013-12-16

**Authors:** Ying Li, Hua Liu, Yasuto Sato

**Affiliations:** 1 Department of Social Medicine, School of Public Health, Zhejiang University, Zhejiang, China; 2 School of Basic Medical Sciences, Zhejiang University, Zhejiang, China; 3 Department of Hygiene and Public Health II, Tokyo Women's Medical University, Tokyo, Japan; Baylor College of Medicine, United States of America

## Abstract

**Objective:**

Although serum C-peptide was previously considered biologically inactive, a growing number of recent studies have shown that it is an active peptide with important physiologic functions. The present study aimed to investigate the association of serum C-peptide level with bone mineral density (BMD) in residents of the United States.

**Methods:**

The study included 6,625 participants aged 12–85 years. Total and regional BMD were measured using dual-energy X-ray absorptiometry. Stratified multiple linear regression analysis was performed to determine the association of the serum C-peptide level with BMD. Three regression models were produced for each stratum. All models were adjusted for ethnicity, height, weight, education level, physical activity, smoking status, alcohol use, triglycerides and creatinine level, and models 2 and 3 were further adjusted for the fasting plasma glucose (FPG) and alkaline phosphatase (ALP) levels, respectively.

**Results:**

Sex-specific results showed a significant association between the serum C-peptide level and total BMD in both sexes. Stratified analyses based on age and body mass index showed that serum C-peptide levels were significantly negatively associated with most regional BMD, and most of these associations remained significant after stratification based on the serum insulin level.

**Conclusion:**

The serum C-peptide level was significantly negatively associated with the total and most regional BMD. These findings suggest that serum C-peptide may have biological activity associated with bone metabolism and therefore serum C-peptide control is advisable in order to reduce the risk of low bone mineral density.

## Introduction

Osteoporosis has important effects on health outcomes, and it has become a common disease worldwide. It is known to be associated with increased morbidity and all-cause mortality [1]–[3]. Low bone mineral density (BMD) is an important risk factor for osteoporosis, and the public health significance of health problems arising from decreased BMD is being increasingly recognized. Many genetic, environmental, and lifestyle factors are known to be associated with BMD [4], [5]. Some studies have shown that BMD increases and the incidence of fractures decreases with obesity; however, other studies have shown negative associations between fat mass and BMD after adjustment for body weight [6], [7]. The body mass index (BMI) is a measure of overweight or obesity, most often used to evaluate the associations among obesity, BMD, diabetes, and insulin secretion. Several clinical studies have shown that diabetes and insulin resistance are closely associated with BMD. Type 2 diabetes has been found to be positively correlated with BMD, but BMD is usually low in diabetic patients with poor glycemic control [8], [9]. However, the BMD is normal or low in patients with type 1 diabetes, and insulin resistance, as measured using the intravenous glucose tolerance test, is correlated positively with BMD [10]. Studies have shown that hyperglycemia can lead directly to a negative calcium balance as well as impair insulin-like growth factor I-induced osteoblast proliferation [11], [12]. Further, clinical studies have shown that the association between insulin exposure and BMD may reflect the direct effects of insulin on bone cells [13].

Both insulin resistance and impaired insulin secretion are known to play important roles in the development of hyperglycemia in patients with type 2 diabetes. Serum C-peptide, which was previously considered an inactive peptide, is known to be a useful marker of beta-cell function. However, a few recent studies suggest that it is an active peptide hormone with important physiologic functions. Serum C-peptide may be involved in glucose transport and the stimulation of microvascular blood flow. Further, studies have shown that the basal C-peptide level is significantly elevated among patients with metabolic syndrome and diabetes. Another recent study reported that the serum C-peptide level is independently associated with cardiovascular disease, cancer, and total mortality [14], [15]. However, currently, the metabolic characteristics of serum C-peptide are receiving considerable attention. Although the serum C-peptide level is commonly considered an index of insulin secretion, it is higher in the elderly than in the young, despite these groups having similar serum insulin levels [16]. To our knowledge, only a single cross-sectional study has evaluated the effect of insulin resistance on BMD using the urine C-peptide as a surrogate marker for endogenous insulin secretion, and this study found that the urine C-peptide level correlated positively with femoral neck BMD in men and postmenopausal women with type 2 diabetes [17]. Nonetheless, the effect of serum C-peptide level on BMD in healthy individuals remains unclear.

The aim of the present study was to investigate the association between the serum C-peptide level and BMD in a large population-based cross-sectional study using data from the National Health and Nutrition Examination Survey (NHANES). The analysis was stratified to ensure that any correlations between the serum C-peptide level and BMD were independent of the insulin level, BMI, and age.

## Materials and Methods

### Subjects

This study was based on the NHANES, a population-based survey that aimed to assess the health and nutritional status of adults and children in the general population of the United States. Clusters of households were studied in 2-year intervals spanning 1999 through 2004, and ≥1 member of each household was selected for the sample. Three representative cross-sectional samples comprising a total of 38,077 US residents were selected through a stratified multistage probability sampling process, of which 31,126 were interviewed and 29,402 were examined. All participants provided written informed consent, if the participant is 17 years or younger, the written informed consent will also be obtained from parental. The study was approved by the institutional review board at the Centers for Disease Control and Prevention (Atlanta, Georgia).

### Information Collection

The cross-sectional survey consisted of 2 parts: a home interview and a health examination. During the home interview, the participants were asked about their general health status, disease history, diet, and physical activity level and were informed that anything that they said during this interview would be confidential. With regard to physical activity, the participants were questioned about general daily activities, leisure time activities, and sedentary activities. The participants reported whether they had engaged in vigorous or moderate-intensity physical activity during the past 30 days. Respondents who answered yes to vigorous or moderate activities, but did not give at least one vigorous or moderate activity, or reported a duration of less than 10 minutes, were recoded to no. Smoking status was classified as never, former, or current smoking. Smokers were asked about the number of cigarettes smoked (per day/week/month/year), the age at which they had started smoking, and the total number of years for which they had smoked. Drinkers were defined as respondents who answered that they had had at least 12 drinks (any type of alcoholic beverage) during the past 12 months. The educational levels were classified as high school or below, high school diploma, and college or above. The health examination was carried out in a mobile examination center. The tests performed were selected on the basis of each participant's age, sex, and current medical conditions.

### Biochemical measurements

Serum c-peptide, serum insulin, and fasting plasma glucose (FPG) levels were measured during the morning examination session only. The participant had fasted for 8 to 24 hours. Serum C-peptide (nmol/L) was measured using the radioimmunoassay method. Since serum obtained from venous blood is required for the procedure, 7 mL venous blood was drawn in a serum clot tube, allowed to clot at room temperature for 20 minutes, and centrifuged in a refrigerated centrifuge at 4°C at 2000×*g* for 10 min. The serum was drawn off and stored in a plastic cryovial at −20°C until it could be transported to the laboratory. Frozen serum specimens had to be delivered within 24 h of collection. In the laboratory, the specimen was logged in and stored at −70°C until analysis. The analytical assay was designed to have a precision of ≤10% of the total coefficient of variation. Serum insulin was measured using the two-site immunoenzymometric assay method with the same sample-collection process as that mentioned above. FPG concentrations were determined using the hexokinase method. A detailed description of the quality assurance and quality control procedures can be found on the NHANES website [18].

### Anthropometrics measurements

The weight, standing height, and waist circumference (WC) were measured according to a standard protocol at the mobile examination center. Height was measured using an electronic stadiometer and weight, using a digital scale connected to the integrated survey information system. The WC was measured using a metal tape. BMI was calculated as the weight in kilograms divided by the square of the height in meters (kg/m^2^).

### Dual energy X-ray absorptiometry measurements

Dual energy X-ray absorptiometry (DXA) is the method most widely used to assess BMD. In the survey, whole-body DXA scans (Hologic QDR 4500A fan beam X-ray bone densitometer; Hologic, Inc., Bedford, Massachusetts) were performed to estimate BMD [19]. All scans were analyzed using Hologic Discovery software version 12.1. The results for each participant were reviewed by the Department of Radiology of the University of California, San Francisco, using standard radiologic techniques. The BMD was obtained for the head, arms, legs, trunk, pelvis, ribs, spine, and total. The BMD of the arms was calculated by adding the individuals' values obtained for the left and right arms. The leg and rib BMD were determined similarly, and the spinal BMD was determined from the values of the thoracic and lumbar spines. Women who reported that they were pregnant at the time of the examination were not scanned. We obtained 16,973 non-missing data sets from 21,230 participants who were eligible for DXA.

### Statistical analysis

We restricted our analysis in the present study to the 16,973 participants for whom complete DXA scan assessment data were available. Of these participants, we excluded participants with missing serum C-peptide measurement values. In addition, we excluded self-reported diabetes and diabetes medication use (n = 422). After the application of these criteria, 6,625 subjects (3,625 men and 3,000 women) were included in the analysis. First, descriptive statistics were applied to describe the general characteristics of the study participants. Sex-specific continuous variables were presented as the mean and standard deviation and categorical variables, as weighted percentages. We calculated the 6-year sample weights as follows: WT1999–2004 = (2/3)×WT1999–2002+(1/3)×WT2003–2004. These sample weights were based on population estimates from the Bureau of the Census [20]. Weighted multiple linear regression analysis was performed to estimate the associations between the serum C-peptide level and the BMD values of the head, arm, leg, trunk, rib, spine, pelvis, and total. Weighted regression adjusting for unequal error variances gives each stratum the same relative importance in the sample as it has in the population. We used subsample weights (serum C-peptide) instead of the full sample mobile examination center weights to compute the weighted regression coefficients: Var(B) = (X'WX)^−1^(X'W2X)(X'WX)^−1^×S^2^, where X is the n×k matrix of the explanatory variables, W is the n×n diagonal matrix of the normalized sample weights, and S^2^ is the estimated variance of the regression error term [21]. The use of subsample weights in the analysis greatly reduces bias caused by missing serum C-peptide measurements. Further, we conducted a stratified analysis to reduce the effects of important confounding factors. The confounding factors were classified as follows: age (<20, 20–39, 40–59, or ≥60 years), sex (male or female), BMI (<25 or ≥25 kg/m^2^), and serum insulin level (<43.14, 43.14–87.36, or >87.36 pmol/L). Each stratified multiple linear regression analysis included 3 separate models adjusted for different sets of variables. In model 1, we adjusted for the subjects' ethnicity, height, weight, education level, physical activity, smoking status, alcohol use, triglycerides and creatinine level in all cases and for age and sex, unless these parameters had been used for stratification. For women, we also adjusted for menopausal status. In models 2 and 3, we further adjusted for FPG and serum alkaline phosphatase (ALP) levels, respectively. All analyses were performed using SAS for Windows (version 9.2).

## Results

The characteristics of the study participants are shown in Table 1. The mean age of the participants was 35.2 years for men and 39.3 years for women. Approximately 72% of the participants were non-Hispanic whites. Further, 56% were classified as overweight or obese (BMI ≥ 25 kg/m^2^) and 34% reported engaging in light or no regular physical activity. The fasting serum C-peptide level did not differ significantly between men and women.

**Table 1:** Characteristics of study participants by sex from National Health and Nutrition Examination Survey (1999–2004).

Variable	Men		Women	
	(n = 3,625)		(n = 3,000)	
Continuous variable (Mean, SD)				
Age (years)	35.2	(21.3)	39.3	(21.9)
Weight (kg)	76.7	(17.7)	68.3	(16.1)
Height (cm)	172.8	(9.0)	161.0	(7.1)
Body mass index (kg/cm^2^)	25.5	(4.9)	26.3	(5.8)
Serum C-Peptide (nmol/L)	0.8	(0.4)	0.8	(0.3)
Total bone mineral density (g/cm^2^)	1.2	(0.1)	1.1	(0.1)
Categorical variable (N, Weighted %)				
Ethnicity				
Non-Hispanic white	1491	(70.8)	1368	(73.1)
Non-Hispanic black	853	(9.9)	626	(10.4)
Mexican-American	996	(9.0)	771	(6.8)
Other Hispanic	146	(5.2)	135	(5.7)
Other	139	(5.1)	100	(4.0)
Education				
Less than high school	1931	(29.6)	1347	(25.6)
High school diploma	620	(23.7)	622	(24.5)
More than high school	1070	(46.7)	1026	(49.9)
Physical activity				
Vigorous	712	(16.5)	355	(12.4)
Moderate	1842	(54.8)	1566	(54.3)
Light or no	1040	(28.7)	1062	(33.3)
Smoking status				
Never	1941	(54.7)	1949	(69.6)
Former	524	(21.7)	300	(14.6)
Current	641	(23.6)	352	(15.8)
Alcohol use				
Yes	2002	(73.7)	1313	(61.5)
No	1377	(26.3)	1477	(38.5)

The description of categorical variable based on non-missing data only.

The results of the multiple regression analysis stratified by sex are shown in Table 2. In men, the serum C-peptide level was significantly negatively associated with the total body BMD values in all 3 models, after adjustment for age, ethnicity, height, weight, education level, physical activity, smoking status, alcohol use, C-reactive protein, triglycerides and creatinine level. In women, after additional adjustment for menopausal status, the serum C-peptide level was found to be negatively associated with body BMD in all models.

**Table 2:** The standardized regression coefficients of serum C-peptide level for total body bone mineral density from multiple regression analysis stratified by sex.

	Total bone mineral density (g/cm^2^)					
Variable	Men			Women		
	Model 1	Model 2	Model 3	Model 1	Model 2	Model 3
Serum C-peptide (nmol/L)	−0.236***	−0.193***	−0.187***	−0.092**	−0.109**	−0.098**
Age (years)	−0.063**	−0.102***	−0.144***	0.220***	−0.206***	−0.199***
Ethnicity	0.065**	0.049	0.069**	0.067**	0.079*	0.059*
Education level	0.012	0.079**	−0.019	0.023	−0.038	−0.011
Smoking status	0.065**	−0.086**	0.061**	0.098***	−0.061	0.069*
Alcohol use	−0.092***	−0.080**	−0.057*	−0.029	−0.020	−0.006
Physical activity	−0.033	−0.025	−0.060**	−0.024	−0.006	−0.016
Weight	0.439***	0.390***	0.370***	0.292***	0.300***	0.340***
Height	0.153***	0.178***	0.149***	0.091***	0.079*	0.057
C-reactive protein (mg/dL)	0.037	−0.014	0.032	−0.034	−0.049	−0.049
Triglycerides (mmol/L)	0.019	−0.003	−0.002	0.077**	−0.002	−0.057*
Creatinine (umol/L)	0.131***	0.124***	0.086***	0.087***	0.053	0.068*
FPG or ALP		0.046	−0.289***		−0.008	−0.243***
Menopausal status				−0.168***	−0.188***	−0.129***

β coefficients are adjusted for age, ethnicity, height, weight, education level, physical activity, smoking status, alcohol use, C-reactive protein, triglycerides and creatinine level. In women, additionally adjusted for menopausal status. Model 1, the

Model 2, additionally adjusted for the fasting plasma glucose.

Model 3, additionally adjusted for the serum alkaline phosphatase.

Slopes of regression at the <0.001 level.

Slopes of regression at the <0.01 level.

Slopes of regression significant at the <0.05 level.

FPG or ALP: fasting plasma glucose or alkaline phosphatase.

Table 3 shows the results of the age-stratified multiple regression analysis. In the <20 years age group, the serum C-peptide level was negatively associated with total BMD in all models (P<0.01 for all, except spine and head BMD in model 3). In the 20–39 years age group, the serum C-peptide level was not significantly associated with spine and pelvic BMD after adjusting for the FPG level. In the 40–59 years age group, the serum C-peptide level was negatively associated with total, arm, leg, trunk and rib BMD. In model 3, after additional adjustment for the ALP level, the spinal, pelvic, and head BMD values were not associated with the serum level of C-peptide in this group. In the ≥60 years age group, no significant association was found between the serum level of C-peptide and BMD; only leg BMD in model 3 showed a significant association with the serum C-peptide level.

**Table 3:** The standardized regression coefficients of serum C-peptide level for total body and regional bone mineral density from multiple regression analysis stratified by age group.

C-peptide	Bone mineral density (g/cm^2^)							
(nmol/L)	Total	Trunk	Arm	Leg	Rib	Spine	Pelvic	Head
<20 years (n = 2318)								
Model 1	−0.138***	−0.152***	−0.122***	−0.106***	−0.147***	−0.098***	−0.111***	−0.079**
Model 2	−0.134***	−0.153***	−0.120***	−0.107***	−0.148***	−0.094***	−0.109***	−0.066**
Model 3	−0.108**	−0.111***	−0.106***	−0.116***	−0.136***	−0.016	−0.066**	−0.023
20–39 years (n = 1570)								
Model 1	−0.174***	−0.166***	−0.160***	−0.142***	−0.149***	−0.125***	−0.081**	−0.066*
Model 2	−0.149***	−0.105**	−0.158***	−0.129***	−0.107**	−0.044	−0.031	−0.057*
Model 3	−0.151***	−0.168***	−0.150***	−0.112***	−0.158***	−0.136***	−0.086**	−0.056
40–59 years (n = 1371)								
Model 1	−0.146**	−0.127***	−0.110***	−0.109***	−0.110***	−0.103**	−0.018	−0.094**
Model 2	−0.132***	−0.100**	−0.114***	−0.116***	−0.101**	−0.069	−0.032	−0.071
Model 3	−0.084*	−0.050*	−0.080**	−0.069*	−0.071*	−0.016	0.044	−0.049
≥60 years (n = 1366)								
Model 1	−0.039	−0.004	−0.038	−0.032	−0.023	0.015	0.024	−0.023
Model 2	−0.026	0.022	−0.039	−0.035	−0.013	0.032	0.047	−0.004
Model 3	−0.048	0.029	−0.059	−0.071*	0.001	0.035	0.054	−0.017

β coefficients are adjusted for gender, ethnicity, height, weight, education level, physical activity, smoking status, alcohol use, triglycerides and creatinine level. Model 1, the

Model 2, additionally adjusted for the fasting plasma glucose.

Model 3, additionally adjusted for the serum alkaline phosphatase.

Slopes of regression significant at the <0.001 level

Slopes of regression significant at the <0.01 level

Slopes of regression significant at the <0.05 level

BMI-stratified multiple regression analysis showed that after the effects of confounding factors such as overweight and obesity were controlled, the serum C-peptide was significantly negatively associated with most regional BMDs (Table 4). In the BMI <25 kg/m^2^ category, the association was not significant with spinal, pelvic, and head BMD after adjusting for the ALP level. In the BMI ≥ 25 kg/m^2^ category, the serum C-peptide level was not associated with only pelvic BMD after adjusting for the FPG or ALP level.

**Table 4:** The standardized regression coefficients of serum C-peptide level for total body and regional bone mineral density from multiple regression analysis stratified by BMI.

C-peptide	Bone mineral density (g/cm^2^)							
(nmol/L)	Total	Trunk	Arm	Leg	Rib	Spine	Pelvic	Head
BMI <25 kg/cm2								
Model 1	−0.133***	−0.102***	−0.125***	−0.115***	−0.111***	−0.047*	−0.027	−0.071***
Model 2	−0.132***	−0.107***	−0.112***	−0.103***	−0.096***	−0.054*	−0.050*	−0.084***
Model 3	−0.073**	−0.065**	−0.074***	−0.055*	−0.058*	−0.005	−0.031	−0.030
BMI ≥25 kg/cm2								
Model 1	−0.139***	−0.115***	−0.124***	−0.106***	−0.114***	−0.086***	−0.048**	−0.094***
Model 2	−0.135***	−0.088***	−0.137***	−0.121***	−0.098***	−0.047*	−0.016	−0.080***
Model 3	−0.112***	−0.050	−0.133***	−0.109**	−0.080**	−0.040*	−0.004	−0.064*

β coefficients are adjusted for age, gender, ethnicity, height, weight, education level, physical activity, smoking status, alcohol use, triglycerides and creatinine level. Model 1, the

Model 2, additionally adjusted for the fasting plasma glucose.

Model 3, additionally adjusted for the serum alkaline phosphatase.

Slopes of regression significant at the <0.001 level.

Slopes of regression significant at the <0.01 level.

Slopes of regression significant at the <0.05 level.

The serum insulin level is considered to be a strong confounder of the association between the serum C-peptide level and BMD. The results of serum insulin-stratified multiple regression analysis showed that serum C-peptide level was negatively associated with BMD, independent of the serum insulin level. In the insulin level <43.14 pmol/L category, the serum C-peptide level was negatively associated with the total and all regional BMDs after adjustments were made for age, sex, ethnicity, height, weight, education level, physical activity, smoking status, alcohol use, triglycerides and creatinine level in model 1. In model 2, no significant association was observed with head BMD after adjustment for the FPG level was made. After additional adjustments were made for the ALP level in model 3, the association with head BMD was not significant. In the insulin level 43.14–87.36 pmol/L category, serum C-peptide level was not associated with rib, spine, and head BMD after adjusting for the ALP level. In the insulin >87.36 pmol/L category, the serum C-peptide level was not associated with spinal, pelvic, or trunk BMD but was significantly associated with the total, arm, and leg BMD (Table 5).

**Table 5:** The standardized regression coefficients of serum C-peptide level for total body and regional bone mineral density from multiple regression analysis stratified by serum insulin level a.

C-peptide	Bone mineral density (g/cm^2^)							
(nmol/L)	Total	Trunk	Arm	Leg	Rib	Spine	Pelvic	Head
Insulin <43.14 pmol/L								
Model 1	−0.133***	−0.146***	−0.109***	−0.091***	−0.111***	−0.135***	−0.083***	−0.072**
Model 2	−0.083**	−0.097**	−0.081***	−0.069**	−0.047	−0.065*	−0.069*	−0.012
Model 3	−0.138***	−0.161***	−0.118***	−0.108***	−0.131***	−0.155***	−0.088**	−0.044
Insulin 43.14–87.36 pmol/L								
Model 1	−0.104***	−0.103***	−0.085***	−0.082**	−0.087***	−0.058*	−0.074***	−0.058*
Model 2	−0.103***	−0.102***	−0.086***	−0.084***	−0.086**	−0.057*	−0.073***	−0.055*
Model 3	−0.059*	−0.059*	−0.051*	−0.061**	−0.043	−0.016	−0.064*	−0.009
Insulin >87.36 pmol/L								
Model 1	−0.090**	−0.026	−0.116***	−0.091***	−0.047	−0.009	−0.024	−0.057
Model 2	−0.099***	−0.029	−0.123***	−0.104***	−0.046	−0.002	−0.029	−0.050
Model 3	−0.082*	−0.004	−0.121***	−0.075*	−0.067	−0.038	−0.047	−0.073

β coefficients are adjusted for age, sex, ethnicity, height, weight, education level, physical activity, smoking status, alcohol use, triglycerides and creatinine level. Model 1, the

Model 2, additionally adjusted for the fasting plasma glucose.

Model 3, additionally adjusted for the serum alkaline phosphatase.

Slopes of regression significant at the <0.001 level.

Slopes of regression significant at the <0.01 level.

Slopes of regression significant at the <0.05 level.

= 4,359.^a^ Fasting serum insulin, n

## Discussion

In this large, national population-based study, the serum C-peptide level was found to be negatively associated with the total and most regional BMDs. The observed association remained significant in analyses stratified by sex, age, BMI, and serum insulin level. Moreover, the negative association between the serum C-peptide level and the BMD was independent of the ethnicity, height, weight, education level, physical activity, smoking status, alcohol use, triglycerides, creatinine, FPG, and ALP levels.

Several previous studies have examined the associations of BMD with obesity, diabetes, insulin resistance, and serum insulin levels [22]–[24]. As noted above, some studies have reported a positive association between BMD and fat mass and an inverse association between osteoporosis and obesity [25], [26]. Similarly, empirical studies have shown that BMD is higher in patients with type 2 diabetes, while others concluded that BMD is lower in patients with diabetes [27]. Obesity is known to be an important risk factor for type 2 diabetes [28], and the serum level of insulin could be responsible, in part, for mediating the putative positive association between type 2 diabetes and higher BMD [29]. In addition, clinical examinations usually use the serum or urine C-peptide level to evaluate insulin resistance and as a surrogate marker of endogenous insulin secretion [30]. One study found the level of C-peptide to be associated with BMI [31]. Yamaguchi et al. evaluated the association between femoral neck BMD and the urine level of C-peptide only as a marker of insulin secretion among patients with type 2 diabetes. Few previous studies have investigated the relationship between BMD and the bioactivity of C-peptide. In the present study, we investigated the independent association between BMD and the serum C-peptide level with an emphasis on the bioactivity of the peptide. Using a stratified design and multivariate adjustment methods, this study showed that the association was independent of the serum insulin concentration and the abovementioned group of complex confounding factors.

The mechanism underlying the association between the serum C-peptide level and BMD in healthy individuals is unclear at present. A cohort study of 51,529 US residents showed that the serum C-peptide level was negatively associated with calcium intake after adjusting for the vitamin D and 25-hydroxyvitamin D levels in women; in men, it was negatively associated with the vitamin D levels after adjusting for BMI. Additionally, some molecular-level studies have shown that C-peptide can increase the intracellular calcium concentration in human cells [32]. The physiological increase in intracellular calcium is important for normal bone cell function [33], [34]. Another study also demonstrated that serum C-peptide could stimulate Na+K+ ATPase activity. Although there are tissue-specific differences in the regulation of Na+K+ ATPase activity, in all cases, the Na+K+ ATPase controls the concentration of free calcium either directly or indirectly, and it was found to be related to osteoclasts in bone metabolism [35], [36].

The age-stratified analysis showed that in the ≥60 years age group, the serum C-peptide level was not negatively associated with any BMD value, except trunk, spinal and pelvic BMD in model 3. We investigated the possible causes for this difference between the ≥60 years age group and the other age groups. Many studies have reported that the serum C-peptide level increases with age in individuals with and without diabetes [37]. These results were similar to our findings: the mean serum C-peptide level was 0.69, 0.71, 0.78, and 0.90 nmol/L in the <20, 20–39, 40–59, and ≥60 years age groups, respectively. Basu et al. also reported that the serum C-peptide level was higher in the elderly than in the young [38]. They found that the peak postprandial serum C-peptide level was also higher, although the increase above the basal level 20 min after a meal was lower, in the elderly than in the young. Previous studies in which the insulin secretion and hepatic insulin clearance rates were calculated using the serum C-peptide model reflected this age-related change in the serum C-peptide level [39], indicating that an increase in hepatic insulin extraction with age may offset the increase in insulin secretion; other studies also reported decreased total insulin clearance and an increase in hepatic insulin extraction in the elderly [40]. Insulin secretion is altered in the elderly, possibly due to an age-related decrease in beta-cell mass [41], [42]. Further, numerous facets of insulin secretion are abnormal in elderly individuals, and the hormonal milieu that commonly accompanies “normal” aging is complex. Therefore, we believe that the lack of a statistically significant association between the serum C-peptide level and BMD in the ≥60 years age group could have been due to age-related changes in the C-peptide level, although the mechanism underlying this effect remains unclear.

Although the present studies have shown a statistically significant association between serum C-peptide levels and total and most regional BMD, the molecular biological mechanisms underlying this relationship require further investigation. Additionally, the association between serum C-peptide levels and BMD was relatively weak compared to that between other risk factors and BMD, such as alkaline phosph alkaline phosphatase or weight. However, the study's findings are significant because of the strength of the correlation between C-peptide and BMD that was observed in this study. Specifically, in the present study, an independent biological correlation was first observed between serum C-peptide and BMD, and this finding has important significance for further studies.

A major strength of the present study is that using stratified multivariate regression analysis, we excluded or controlled for many important confounding factors, including age; sex; ALP, FPG, and serum insulin levels; and BMI. In addition, to our knowledge, this is the first study to explore the serum C-peptide level as an independent risk factor for BMD. Although the relationships among BMD and obesity, diabetes, and insulin secretion are very complex and the effects of these inter-related factors on bone metabolism are still not fully clear, the present study indicates that BMD status can be explained, at least in part, by the serum C-peptide level. Further research is needed to clarify the mechanism underlying this association. In addition, our results are based on a national survey, and the analysis was enabled by highly representative biomarker data.

This study also has several limitations that must be acknowledged. First, because of its cross-sectional design, we could only determine the association between serum C-peptide and BMD and could not determine causality. Second, the study used only a single measurement. Third, there is lack of information about the measurement of serum C-peptide, despite we use the provided sample weight to minimize the effect. Additionally, we were unable to investigate the effects of the interaction among various risk factors on BMD. Although the inclusion of a large number of records with no missing data allowed us to consider many of the most relevant risk factors in our analysis, the sample size was not adequately large to allow evaluation of the interactions among various risk factors.

In conclusion, our results indicate that the serum C-peptide level is significantly negatively associated with BMD. Unlike in other studies, this study found that the association between serum C-peptide and BMD was independent of the serum insulin level, and the serum C-peptide level was not used as an indicator of beta-cell function. Our results suggest that serum C-peptide may directly affect BMD or bone metabolism. These findings have important clinical and public health implications. However, prospective basic and clinical studies are needed to validate the current findings further and explore the mechanisms underlying the association between the serum C-peptide level and BMD.
